# Supplementary material for: Evidence supported by Mendelian randomization: impact on inflammatory factors in knee osteoarthritis
Source: Front Med (Lausanne). 2024 May 28;11:1382836. doi: 10.3389/fmed.2024.1382836 (PMC11165061; doi:10.3389/fmed.2024.1382836)
Supplement: Supplementary file 1 [file Data_Sheet_1.ZIP › data/GCST90274804_5e6/804.leaveoneout.pdf]

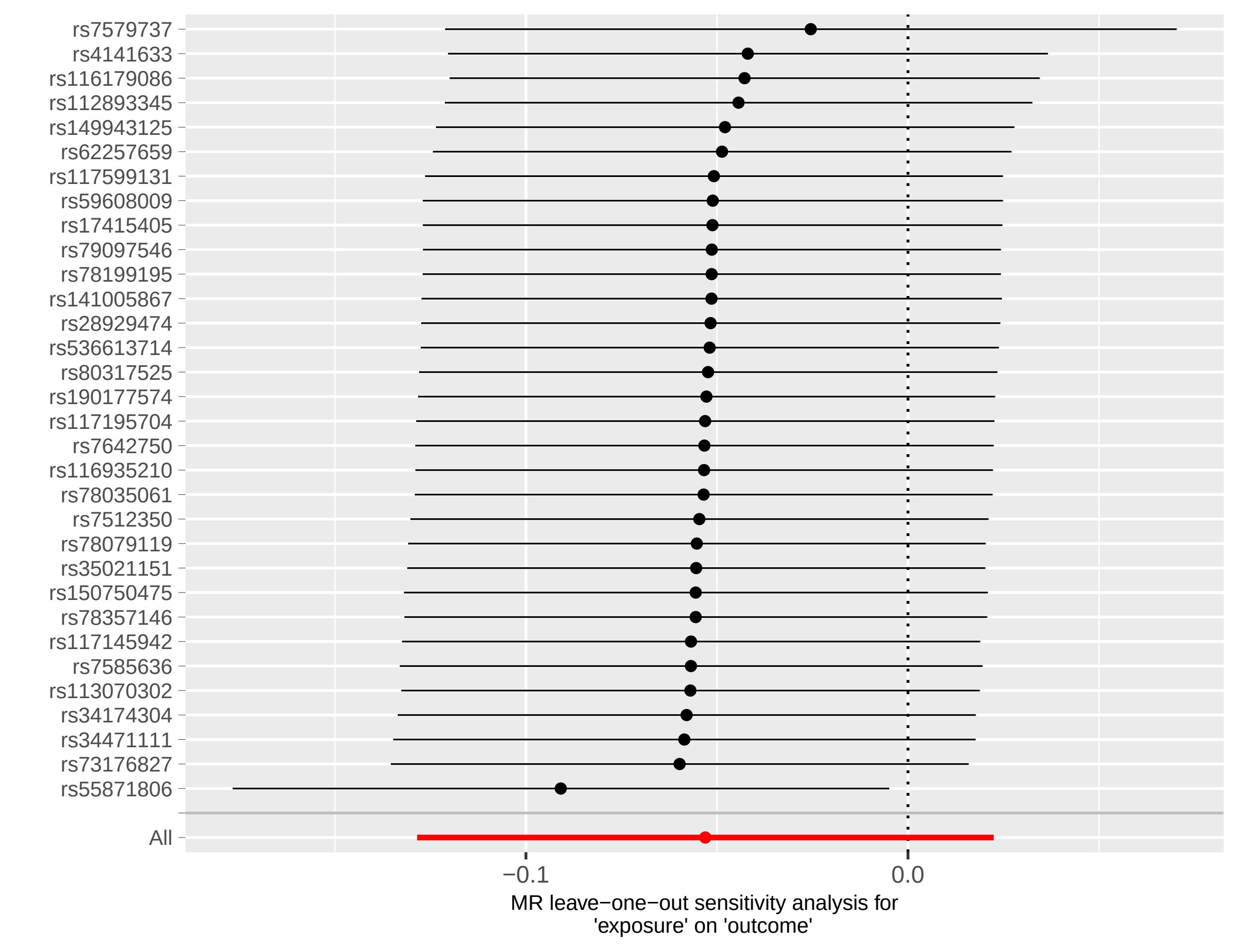
rs7579737
rs4141633
rs116179086
rs112893345
rs149943125
rs62257659
rs117599131
rs59608009
rs17415405
rs79097546
rs78199195
rs141005867
rs28929474
rs536613714
rs80317525
rs190177574
rs117195704
rs7642750
rs116935210
rs78035061
rs7512350
rs78079119
rs35021151
rs150750475
rs78357146
rs117145942
rs7585636
rs113070302
rs34174304
rs34471111
rs73176827
rs55871806
All
−0.1
0.0
MR leave−one−out sensitivity analysis for
'exposure' on 'outcome'
